# Standardization of the infusion sequence of antineoplastic drugs used in the treatment of breast and colorectal cancers

**DOI:** 10.1590/S1679-45082018RW4074

**Published:** 2018-05-25

**Authors:** Amanda Alves da Silva, Juliane Carlotto, Inajara Rotta

**Affiliations:** 1 Setor de Farmácia Hospitalar, Complexo Hospital de Clínicas, Universidade Federal do Paraná, Curitiba, PR, Brazil.

**Keywords:** Administration, intravenous, Antineoplastic agents/administration & dosage, Breast neoplasms, Colorectal neoplasms, Administração intravenosa, Antineoplásicos/administração & dosagem, Neoplasias da mama, Neoplasias colorretais

## Abstract

The definition of antineoplastic administration sequences can help planning of therapeutic regimens in a more rational way, and thus optimize chemotherapy effects on patients, increasing efficacy and reducing toxic effects. In this way, this study aimed to evaluate the infusion order of antineoplastic agents of the main therapeutic protocols used in the treatment of colorectal and breast cancer which are used in a tertiary hospital, identifying possible interactions dependent on the infusion sequence. For the definition of protocols adopted in the hospital, medical prescriptions were used in the period of January to March 2016 and a literature review was conducted to search for studies assessing the sequence of administering the selected regimens. The databases used were SciELO, LILACS and MEDLINE, in addition to Micromedex Solutions^®^ and UpToDate^®^. A total of 19 protocols were identified for antineoplastic therapy, 11 for colorectal cancer and 8 for breast cancer. The selected articles provided evidence for administration order of 19 protocols, and three protocols did no report relevance of infusion sequence. Sequence-dependent interactions were mainly related to toxicity, pharmacokinetics and efficacy of the drug combination. The definition of the infusion sequence has a great impact on the optimization of therapy, increasing efficacy and safety of the protocols containing combined antineoplastic therapies.

## RESUMO

A definição de sequências de administração de antineoplásicos pode proporcionar o planejamento dos esquemas terapêuticos de forma mais racional e, assim, otimizar o efeito da quimioterapia nos pacientes, aumentando a eficácia e reduzindo o aparecimento de efeitos tóxicos. Desta forma, o objetivo deste estudo foi avaliar a ordem de infusão dos antineoplásicos constituintes dos principais protocolos terapêuticos para o tratamento dos cânceres de mama e colorretal utilizados em um hospital terciário, identificando possíveis interações dependentes da sequência de infusão. Para definição dos protocolos adotados na Instituição, foram utilizadas as prescrições no período de janeiro a março de 2016, sendo então realizada uma revisão de literatura, para buscar estudos que avaliaram a sequência de administração dos esquemas selecionados. Para tanto, as seguintes bases de dados foram utilizadas: SciELO, LILACS e MEDLINE, além das plataformas Micromedex Solutions^®^ e UpToDate^®^. Foram identificados 19 protocolos para terapia antineoplásica, sendo 11 para câncer colorretal e 8 para câncer de mama. Os artigos selecionados forneceram evidências para ordem de administração de 19 protocolos, e em 3 protocolos, não foi evidenciada a relevância da sequência infusional. As interações dependentes de sequência foram principalmente relacionadas à toxicidade, farmacocinética e eficácia da combinação de fármacos. A definição da sequência infusional possui grande impacto na otimização da terapia, aumentando a eficácia e a segurança dos protocolos, contendo terapias combinadas de antineoplásicos.

## INTRODUCTION

Cancer is a worldwide public health issue. In 2012, there were 14.1 million cases of cancer around the world, and it is estimated this number will reach 24 million, in 2025.^(^ ^1^ ^)^ According to the *Instituto Nacional de Câncer “José Alencar Gomes da Silva”* (INCA), in 2016/2017, Brazil had approximately 600 thousand new cases of cancer; in that, breast (28.1%) and colorectal cancer (8.6%) were the most prevalent among women, and prostate (28.6%), tracheal, bronchial and lung (8.1%), and colorectal (7.8%), in males. Non-melanoma skin cancer was not included in this estimate and is the most prevalent in both sexes.^(^ ^2^ ^)^ Colorectal cancer is among the five most frequent neoplasms, and its incidence is not homogeneous throughout Brazil; it is more prevalent in the South and Southeast regions of the country.^(^ ^3^ ^)^

The combination of several drugs with different mechanisms of action is an effective strategy in cancer treatment and provides many benefits. First, the association of two or more drugs with different mechanisms of actions can delay cell mutations and the process of adjustment to cancer. Second, the synergistic effect of the drugs, that is, the combined action of medications leading to potentiated biological effect.^(^ ^4^ ^)^ The pharmacological mechanisms involved in the process of interactions among intravenous solutions are basically pharmacokinetic interactions (involving factors that accelerate or delay absorption, distribution, metabolization and elimination of the drugs used) and pharmacodynamic interactions (factors leading to dysfunction in the pharmacological receptor binding).^(^ ^5^ ^)^ Several antineoplastic agents (*e.g*. doxorubicin, docetaxel, paclitaxel, etc.) are metabolized via the cytochrome P450 pathway (CYP), and other chemotherapeutic drugs (*e.g.* taxanes and platinum agents) present high levels of protein binding. Moreover, many chemotherapeutic drugs have specific mechanisms of action during the cell cycle, and can increase cytotoxicity or antagonize the mechanism of the second agent.^(^ ^6^ ^)^

Recognizing these pharmacokinetic interactions between drugs is important to optimize doses of cytotoxic agents in combined chemotherapy. Many drugs called “cell cycle specific drugs” (CCS) are effective against cancer and act on cells that are in the cell cycle. A second group of agents called “cell cycle nonspecific drugs” (CCNS) are able to kill tumor cells, regardless of their being in the cell cycle or at rest.^(^ ^7^ ^)^ Regarding the infusion order, if the specific antineoplastic agents are administered before nonspecific cycles, maximized effect in cells with high cell division rates, such as neoplastic cells, is theoretically expected. The reason is when the cell cycle is interrupted at the time of division, nonspecific agents can more easily act in the DNA.^(^ ^8^ ^)^ Furthermore, it is argued that it is preferable to first administer the vesicant antineoplastic drug, considering vascular integrity decreases with time. It is therefore advantageous to infuse the vesicant antineoplastic agent when the vein is more stable and less irritated.^(^ ^9^ ^)^

As a focus of this study, we have chosen to work with protocols used for treatment of breast and colorectal cancers, for being the most prevalent in women (breast and colorectal) and are among the most prevalent in men (colorectal), especially in the South and Southeast regions of Brazil.

Even though many reports have been published on this topic, there are no studies containing the evaluation of infusion sequence for all protocols used in antineoplastic therapy. Therefore, to define the infusion sequence, we must often rely on pharmacokinetic and pharmacodynamic evaluations and on drug characteristics. In the literature, there is not yet one single source compiling the ideal administration sequences for the main protocols used in antineoplastic therapy.

## OBJECTIVE

To identify interactions that depend on the infusion sequence to establish recommendations for the administration of antineoplastic agents in protocols used in breast and colorectal cancer treatments.

## METHODS

This is a literature review study about the evaluation of infusion sequence of antineoplastic drugs in protocols used in colorectal and breast cancer treatments, employed in a tertiary hospital in the city of Curitiba (State of Paraná).

To define therapeutic protocols containing antineoplastic drug combinations used at the organization, we analysed medical prescriptions of adult patients seen at the oncology outpatient clinic. We selected prescriptions given between January and March 2016 that included the combinations of antineoplastic drugs used in breast and colorectal cancer treatment. We excluded prescriptions containing regimens in which the antineoplastic drugs had not been administered on the same day, or those that included only one antineoplastic agent, since the objective of the study was to determine the medication infusion sequence.

Based on the therapeutic protocols selected from medical prescriptions, we conducted a literature review to search for studies evaluating the administration sequence of the selected regimens. To that end, we used the databases Scientific Electronic Library Online (SciELO), Latin American and Caribbean Health Sciences Literature (LILACS) and MEDLINE (PubMed). The keywords to identify studies related to the theme included any combination of the protocol name and/or antineoplastic drug, with the English words “administration”, “sequencing” or “interactions”. Additionally, we used the software Micromedex Solutions^®^ and UpToDate^®^ to collect information on the drugs and on possible drug interactions. We included all articles about protocols of interest, giving preference to those conducted in humans and excluding the studies containing protocols of drugs not administered on the same day of treatment.

For situations without a clearly established infusion sequence or with disagreement among authors, we evaluated pharmacokinetics, pharmacodynamics and characteristics of each antineoplastic drug to define the most appropriate sequence. This study was approved by the Research Ethics Committee, protocol 1.776.532, CAAE: 59998016.0.0000.0096.

## RESULTS

A total of 408 prescriptions containing treatment protocols for colorectal and breast cancer were evaluated. Most patients were female (65.8%) and mean age was 53.56 years.

Table 1 shows therapeutic protocols used at the organization for breast and colorectal cancer with the respective number of patients for whom they were prescribed.

**Table 1:** Therapeutic protocols

Protocols	n (%)
AC*	48 (27.53)
FLOX†	19 (10.67)
B-FOL†	18 (10.11)
Cisplatin + gemcitabine	17 (9.55)
MAYO^‡^	15 (8.43)
TC§	10 (5.62)
Cisplatin + irinotecan	9 (5.06)
Fluorouracil + folinic acid	7 (3.93)
Gemcitabine + docetaxel	7 (3.93)
FOLFIRI¶	6 (3.37)
FOLFOX||	5 (2.81)
IFL¶	2 (1.12)
MC DONALD^‡^	2 (1.12)
Paclitaxel + pamidronate	2 (1.12)
M-FOLFOX	2 (1.12)
Paclitaxel + zoledronic acid	2 (1.12)
Docetaxel + pamidronate	1 (0.56)
Trastuzumab + pertuzumab	1 (0.56)
CAF#	1 (0.56)
Cisplatin + paclitaxel	1 (0.56)
FOLFOXIRI**	1 (0.56)
Trastuzumab + paclitaxel	1 (0.56)

* Protocol AC was composed of doxorubicin and cyclophosphamide;

† protocols FLOX and B-FOL were composed of oxaliplatin, folinic acid and fluorouracil, in different combination regimens; protocols MAYO and MC DONALD included folinic acid and fluorouracil;

§ protocol TC was composed of docetaxel and cyclophosphamide;

¶ protocols FOLFIRI and IFL comprised irinotecan, folinic acid and fluorouracil in different combination regimens;

|| protocol FOLFOX was composed of oxaliplatin, folinic acid and fluorouracil;

# protocol CAF comprised doxorubicin, fluorouracil and cyclophosphamide;

** protocol FOLFOXIRI included irinotecan, oxaliplatin, folinic acid and fluorouracil.

In total, 178 patients used 22 different protocols containing a combination of antineoplastic drugs.

The bibliographic search yielded 36 articles that were the basis to evaluate these protocols. The selected articles brought some evidence towards the administration sequence of 19 protocols (Table 2), while for 3 protocols there was no evidence of relevance in the infusion sequence (Table 3). Interactions dependent on the sequence were mainly related to toxicity, pharmacokinetics and efficacy of drug combination.

**Table 2:** Suggested sequence for the therapeutic protocol and reasons for infusion order

Protocol	Suggested sequence Agent				Reasons for suggestion
	First	Second	Third	Fourth	
AC	Doxorubicin CCNS^(^ ^7^ ^)^ Vesicant^(^ ^9^ ^)^	Cyclophosphamide CCNS^(^ ^7^ ^)^	-	-	Compatible in Y^(^ ^10^ ^)^ Cyclophosphamide is a prodrug catalyzed directly by cytochrome P450, mainly through CYP2B6.^(^ ^11^ ^)^ Doxorubicin is a (moderate) inhibitor of the same enzyme.^(^ ^12^ ^)^ To avoid delayed plasma clearance of doxorubicin due to cyclophosphamide metabolism, it is recommended to infuse doxorubicin before cyclophosphamide.^(^ ^5^ ^)^
CAF	Doxorubicin CCNS^(^ ^7^ ^)^ Vesicant^(^ ^9^ ^)^	Fluorouracil CCS^(^ ^7^ ^)^	Cyclophosphamide CCNS^(^ ^7^ ^)^	-	Compatible in Y^(^ ^10^ ^)^ Fluorouracil acts in specific cell cycle phases, while doxorubicin and cyclophosphamide are CCNS.^(^ ^7^ ^)^ However, doxorubicin has a high tissue vesicant potential, which reinforces the importance of it being administered first.^(^ ^9^ ^)^ Cyclophosphamide is a prodrug catalyzed directly by cytochrome P450, especially through CYP2B6.^(^ ^11^ ^)^ Doxorubicin is a (moderate) inhibitor of the same enzyme.^(^ ^12^ ^)^ To avoid delayed plasma clearance of doxorubicin due to cyclophosphamide metabolism, it is recommended to infuse doxorubicin before cyclophosphamide.^(^ ^5^ ^)^ Regarding fluorouracil and cyclophosphamide, it has been observed that fluorouracil sensitizes the DNA so it can be attacked by alkylating agents.^(^ ^13^ ^)^
Cisplatin + Gemcitabine	Gemcitabine CCS^(^ ^7^ ^)^	Cisplatin CCNS^(^ ^7^ ^)^	-	-	Compatible in Y^(^ ^10^ ^)^ Gemcitabine is a CCS drug while cisplatin is CCNS, which justifies this infusion sequence. Also, gemcitabine administration (4 or 24 hours) before cisplatin administration proved less toxic, causing less leukopenia.^(^ ^14^ ^)^
Cisplatin + irinotecan	Cisplatin CCNS^(^ ^7^ ^)^	Irinotecan CCS^(^ ^7^ ^)^	-	-	Compatible in Y^(^ ^10^ ^)^ Irinotecan is a CCS drug, while cisplatin is CCNS. However, with previous administration of cisplatin, there is an increase in the synergistic effect, with the presence of toxicity regardless of the administration sequence.^(^ ^15^ ^,^ ^16^ ^)^
Cisplatin + paclitaxel	Paclitaxel CCS^(^ ^7^ ^)^	Cisplatin CCNS^(^ ^7^ ^)^	-	-	Compatible in Y^(^ ^10^ ^)^ Paclitaxel is a CCS drug, whereas cisplatin is CCNS, which justifies this infusion sequence.^(^ ^7^ ^)^ When cisplatin is administered first, paclitaxel clearance is reduced, and myelosuppression is more severe. It has been suggested that this decrease in paclitaxel clearance, after cisplatin, may be due to cytochrome P450 inhibition, which is responsible for paclitaxel metabolism.^(^ ^17^ ^)^
Docetaxel + pamidronate	Docetaxel CCS^(^ ^7^ ^)^	Pamidronate	-	-	Compatible in Y^(^ ^10^ ^)^ No studies were found on administration order. The recommendation is to administer docetaxel first, considering pamidronate may cause nephrotoxicity, which manifests as nephritic syndrome, kidney function deterioration and renal failure, which could alter docetaxel excretion.^(^ ^18^ ^)^
FOLFIRI	Irinotecan CCS^(^ ^7^ ^)^ + Folinic acid (60 minutes prior)	Fluorouracil CCS^(^ ^7^ ^)^ (bolus)	Fluorouracil CCS^(^ ^7^ ^)^ (continuous infusion – 46 hours)	-	Incompatible in Y (irinotecan – fluorouracil)^(^ ^10^ ^)^ Folinic acid stabilizes thymidylate synthase when administered before fluorouracil, increasing the efficacy and cytotoxicity of the latter.^(^ ^19^ ^,^ ^20^ ^)^ Moreover, for better action of folinic acid, a minimum 60-minute period is required for drug distribution and intracellular metabolism.^(^ ^5^ ^)^ We observed a synergistic effect when there was previous exposure to irinotecan, intensifying fluorouracil-induced DNA damage.^(^ ^21^ ^)^ According to a study by Falcone et al., toxicity was affected by the administration sequence of irinotecan and fluorouracil, with acceptable toxicity when irinotecan was followed by fluorouracil.^(^ ^22^ ^)^ To provide faster patient care, the concomitant initial infusion of irinotecan and folinic acid was proposed, followed by fluorouracil in bolus and fluorouracil in continuous infusion.^(^ ^8^ ^)^ It is worth mentioning the importance of cleaning the Y system between the infusions of irinotecan and fluorouracil, due to incompatibility.
FOLFOXIRI	Irinotecan CCS^(^ ^7^ ^)^	Oxaliplatin CCNS^(^ ^7^ ^)^ + Folinic acid (60 minutes prior)	Fluorouracil CCS^(^ ^7^ ^)^ (continuous infusion–46 hours)	-	Incompatible in Y (irinotecan – fluorouracil)^(^ ^10^ ^)^ Not tested in Y (fluorouracil – oxaliplatin)^(^ ^10^ ^)^ Irinotecan in a prodrug and thus requires hepatic microsomal activation. Therefore, whenever possible, this type of drug should be prioritized in multiple infusions.^(^ ^5^ ^)^ Also, some studies demonstrated there may be an increase in cholinergic side effects of irinotecan when administered after oxaliplatin.^(^ ^23^ ^)^ Dodds et al., observed the cholinergic effects of irinotecan are manifested by inhibiting acetylcholinesterase.^(^ ^24^ ^)^ Similarly to other alkylating agents, oxaliplatin can also inhibit this enzyme, which can potentiate the cholinergic effects of irinotecan. Regarding the combination between oxaliplatin and fluorouracil, Qin et al. observed *in vitro* synergistic effect, *i.e*., apoptosis was more prominent when cells were treated with oxaliplatin first and then with fluorouracil.^(^ ^25^ ^)^ Furthermore, some studies demonstrated oxaliplatin can inhibit the main enzyme of fluorouracil metabolism (dihydropyrimidine dehydrogenase).^(^ ^26^ ^)^ A synergistic effect was observed when there was previous exposure to irinotecan, intensifying DNA damage induced by fluorouracil.^(^ ^21^ ^)^ According to a study by Falcone et al., toxicity was affected by the administering sequence of irinotecan and fluorouracil, with acceptable toxicity when irinotecan was followed by fluorouracil.^(^ ^22^ ^)^ Folinic acid stabilizes thymidylate synthase when administered before fluorouracil, increasing efficacy and cytotoxicity of the latter.^(^ ^19^ ^,^ ^20^ ^)^ Moreover, for better action of folinic acid, a minimum 60-minute period is required for the distribution of the drug and intracellular metabolism.^(^ ^5^ ^)^ It is worth mentioning the importance of cleaning the Y system between the infusions of irinotecan and fluorouracil, due to incompatibility.
FLOX/B-FOL	Oxaliplatin CCNS^(^ ^7^ ^)^	Folinic acid (60 minutes prior)	Fluorouracil CCS^(^ ^7^ ^)^	-	Not tested in Y (fluorouracil – oxaliplatin).^(^ ^10^ ^)^ Folinic acid stabilizes thymidylate synthase when administered before fluorouracil, increasing efficacy and cytotoxicity of the latter.^(^ ^19^ ^,^ ^20^ ^)^ Moreover, for better action of folinic acid, a minimum 60-minute period is required for distribution of the drug and intracellular metabolism.^(^ ^5^ ^)^ Regarding the combination between oxaliplatin and fluorouracil, Qin et al. observed *in vitro* synergistic effect, *i.e*., apoptosis was more prominent when cells were treated with oxaliplatin first and then with fluorouracil.^(^ ^25^ ^)^ Furthermore, some studies demonstrated that oxaliplatin can inhibit the main enzyme of fluorouracil metabolism (dihydropyrimidine dehydrogenase).^(^ ^26^ ^)^
FOLFOX	Oxaliplatin CCNS^(^ ^7^ ^)^ + Folinic acid (60 minutes prior)	Fluorouracil CCS^(^ ^7^ ^)^ (bolus)	Fluorouracil CCS^(^ ^7^ ^)^ (continuous infusion – 46 hours)		Not tested in Y (fluorouracil – oxaliplatin).^(^ ^10^ ^)^ Folinic acid stabilizes thymidylate synthase when administered before 5-FU, increasing the efficacy and cytotoxicity of the latter.^(^ ^19^ ^,^ ^20^ ^)^ Moreover, for better action of the folinic acid, a minimum 60-minute period is required for distribution of the drug and intracellular metabolism.^(^ ^5^ ^)^ Regarding the combination between oxaliplatin and 5-FU, Qin et al., observed *in vitro* synergistic effect; apoptosis was more prominent when cells were treated with oxaliplatin first and then with 5-FU.^(^ ^25^ ^)^ Furthermore, some studies demonstrated oxaliplatin can inhibit the main enzyme of 5-FU metabolism (dihydropyrimidine dehydrogenase).^(^ ^26^ ^)^ To provide more efficient patient care, concomitant initial infusion of oxaliplatin and folinic acid was proposed, followed by fluorouracil in bolus and fluorouracil in continuous infusion.^(^ ^27^ ^)^
IFL	Irinotecan CCS^(^ ^7^ ^)^	Folinic acid (60 minutes prior)	Fluorouracil CCS^(^ ^7^ ^)^	-	Incompatible in Y (irinotecan – fluorouracil).^(^ ^10^ ^)^ Folinic acid stabilizes thymidylate synthase when administered before fluorouracil, increasing the efficacy and cytotoxicity of the latter.^(^ ^19^ ^,^ ^20^ ^)^ Moreover, for better action of the folinic acid, a minimum 60-minute period is required for distribution of the drug and intracellular metabolism.^(^ ^5^ ^)^ A synergistic effect was observed when there was previous exposure to irinotecan, intensifying DNA damage induced by fluorouracil.^(^ ^24^ ^)^ According to a study by Falcone et al., toxicity was affected by the administering sequence of irinotecan and fluorouracil, with acceptable toxicity when irinotecan was followed by fluorouracil.^(^ ^22^ ^)^ It is worth mentioning the importance of cleaning the Y system between the infusions of irinotecan and fluorouracil, due to incompatibility.
MAYO/ McDonald/ fluorouracil + folinic acid	Folinic acid (60 minutes prior)	Fluorouracil CCS^(^ ^7^ ^)^	-	-	Compatible in Y^(^ ^10^ ^)^ Folinic acid stabilizes thymidylate synthase when administered before fluorouracil, increasing the efficacy and cytotoxicity of the latter.^(^ ^19^ ^,^ ^20^ ^)^ Moreover, for better action of the folinic acid, a minimum 60-minute period is required for distribution of the drug and intracellular metabolism.^(^ ^5^ ^)^
Paclitaxel + zoledronic acid	Paclitaxel CCS^(^ ^7^ ^)^	Zoledronic acid	-	-	Compatible in Y^(^ ^10^ ^)^ Increase of synergistic effects due to increased apoptosis.^(^ ^28^ ^)^
Paclitaxel + pamidronate	Paclitaxel CCS^(^ ^7^ ^)^	Pamidronate	-	-	Compatible in Y^(^ ^10^ ^)^ No studies were found describing an administration sequence. The recommendation is to administer paclitaxel first, considering pamidronate can cause nephrotoxicity, which manifests as nephritic syndrome, kidney function deterioration and renal failure, which could alter paclitaxel excretion.^(^ ^18^ ^)^
TC	Docetaxel CCS^(^ ^7^ ^)^	Cyclophosphamide CCNS^(^ ^7^ ^)^	-	-	Compatible in Y^(^ ^10^ ^)^ Docetaxel is a CCS drug, while cyclophosphamide is a CCNS drug, which justifies this infusion sequence. Cyclophosphamide is a prodrug, catalyzed directly through cytochrome P450.^(^ ^11^ ^)^ Docetaxel is also oxidated by cytochrome P450 enzymes, especially by CYP3A4 in the liver.^(^ ^29^ ^)^ We found a few controversial studies that relate ifosfamide and docetaxel because ifosfamide and cyclophosphamide are similar.^(^ ^30^ ^)^ Schijvers et al. observed the AUC of ifosfomide and its metabolites were smaller when docetaxel was administered first.^(^ ^31^ ^)^ A study by Ando et al., suggests that docetaxel can competitively inhibit the biotransformation of the prodrug ifosfamide through the isoenzyme CYP3A4 of cytochrome P450.^(^ ^32^ ^)^ However, the studies we found present no clear evidence for an administration sequence of these drugs.
Trastuzumab + paclitaxel	Trastuzumab	Paclitaxel CCS^(^ ^7^ ^)^	-	-	Compatible in Y^(^ ^10^ ^)^ Lee et al., observed pre-treatment with trastuzumab resulted in better sensitization of breast cancer cells, *i.e*., trastuzumab followed by paclitaxel increased the activation and induction of programmed cell death or cell apoptosis.^(^ ^33^ ^)^ Moreover, due to the possible infusion reaction of the monoclonal antibody, it is recommended that trastuzumab be infused first.^(^ ^34^ ^,^ ^35^ ^)^

CCNS: cell-cycle nonspecific; CCS: cell-cycle specific; AUC: area under the curve.

**Table 3:** Combination of chemotherapeutic agents in which sequence has no effect on efficacy or toxicity

Carboplatin + paclitaxel^(^ ^36^ ^,^ ^37^ ^)^
Gemcitabine + docetaxel^(^ ^38^ ^,^ ^39^ ^)^
Trastuzumab + pertuzumab^(^ ^40^ ^-^ ^42^ ^)^

## DISCUSSION

There are few studies in the literature evaluating the infusion sequence of antineoplastic drugs in chemotherapy protocols. The definition of more adequate administration sequences, considering the constituent drugs, can help planning of therapeutic regimens in a more rational way, and thus optimize chemotherapy effects on patients, increasing efficacy and reducing toxic effects. The main criteria to be considered when defining the ideal order are: pharmacokinetics/pharmacodynamics (including the cell cycle phase in which the antineoplastic drugs act) and drug characteristics (vesicant/irritant and incompatibilities). Moreover, considering that the main target is cell division, the drugs affect all normal tissues in quick division, and they will probably produce some toxicity, either at high or low levels.^(^ ^43^ ^)^

Based on information related to each drug’s pharmacokinetics and pharmacodynamics, it is possible to evaluate antagonism or synergism, in relation to efficacy and safety. The synergistic effect is one of the main reasons for using combined chemotherapy. Synergy is defined as an expected additive effect when individual drugs are combined. Antagonism is when the combination of two drugs reduces or nullifies the effects of one or both. The combination of folinic acid (LV) and fluorouracil (5FU) is an example of two drugs that synergistically act on the same target, yielding antitumoral results.^(^ ^44^ ^)^ Folinic acid increases the effect of 5FU by stabilizing the binding of its converted form (fluorodeoxyuridine acid) to thymidylate synthase, contributing to inhibition of this important enzyme in DNA repair and replication.^(^ ^19^ ^,^ ^20^ ^)^

Among the effects related to the pharmacokinetics ofthe drugs studied, the most frequent were related to metabolism/biotransformation and antineoplastic medication elimination/excretion. Although some drugs are metabolized in their place of absorption, the primary metabolism site is the liver, especially in the P450 cytochrome enzyme complex. Drugs, food and some herbs can induce or inhibit enzymes involved in drug metabolism.^(^ ^45^ ^)^ Therefore, it is important to understand the role of enzymes and transporters in the metabolism of antineoplastic agents and the mechanisms through which they modulate their expression and activity.

Enzyme inhibition usually leads to an increase in the metabolic rate and in the serum concentration of the drug, which can result in increased therapeutic response or toxicity. Enzyme inhibition effects are relatively fast, with their initial effects appearing in 24 hours. Therefore, patients must be frequently monitored.^(^ ^46^ ^)^ A well-known example of drug interaction due to enzyme inhibition is the one between cyclophosphamide and doxorubicin. Cyclophosphamide is a substrate of the enzyme CYP2B6, which means it is a prodrug and requires biotransformation to produce its pharmacologically active cytotoxic compounds. Doxorubicin is a (moderate) inhibitor of this same enzyme. Therefore, CYP inhibitors can reduce the metabolism of substrates of this pathway, leading to decreased serum concentration of cyclophosphamide.^(^ ^12^ ^)^

Most antineoplastic drugs affect mainly cell division. In many cases, the antiproliferative action of antineoplastic drugs is directly in the DNA, resulting in permanent damage and initiating cell apoptosis. A better result of these antineoplastic drugs can be obtained through the action during the S phase of the cell cycle, when the cell is synthetizing a new DNA.^(^ ^44^ ^)^ Thus, regarding infusion order, if we administer a CCS antineoplastic drug before a CCNS agent, we can theoretically expect a maximization of the effects on cells with a high cell division rate, such as the neoplastic cells. Thus, with an interrupted cell cycle, antineoplastic agents can more easily act in the DNA.

In addition, the higher the exposure to antineoplastic drugs, the less stable and more fragile veins become. As a consequence, drugs administered last have a higher chance of leakage, regardless of the technique employed. It is better to administer the vesicant antineoplastic drug first, when the vein is more stable and less irritated. Another possibility is using the “sandwich technique”: a non-vesicant antineoplastic drug first, then the vesicant drug, and lastly another non-vesicant. However, vein integrity decline due to successive cytotoxic cycles suggests vesicant drugs are safer when administered first.^(^ ^47^ ^)^ Therefore, administering vesicant drugs first proves more advantageous and safer for patients.

The limitations of this study include the fact that, in some cases, the infusion sequence was not clearly described in the literature, such as: a) the combination of pamidronate and paclitaxel or docetaxel – the infusion order was based on the fact that pamidronate can cause nephrotoxicity, which could alter the excretion of the other drugs; and b) the combination of cyclophosphamide and docetaxel, whose data were extrapolated from studies with ifosfomide, which belongs to the same drug class of cyclophosphamide.

Nevertheless, this review showed that many infusion sequences have already been well-defined in the literature, regarding safety, efficacy, prevention of excessive toxicity or reduced efficacy. Therefore, understanding the potential drug interactions, medical teams can minimize risks by prescribing adequate drugs with an appropriate infusion order and monitoring signs of interactions.

## CONCLUSION

Defining an infusion sequence greatly optimizes treatment. Such sequences must be based on studies (preferably carried out in humans, about specific sequence evaluations), and pharmacokinetics, pharmacodynamics and drug properties (vesicant drugs or incompatibilities). By means of this research, we propose increasing efficacy and safety of protocols that include combined antineoplastic treatments, and standardizing the administration sequences.
